# Pharmacological Inhibition of Amyloidogenic APP Processing and Knock-Down of APP in Primary Human Macrophages Impairs the Secretion of Cytokines

**DOI:** 10.3389/fimmu.2020.01967

**Published:** 2020-09-03

**Authors:** Philipp Spitzer, Matthias Walter, Caroline Göth, Timo Jan Oberstein, Philipp Linning, Hans-Joachim Knölker, Johannes Kornhuber, Juan Manuel Maler

**Affiliations:** ^1^Department of Psychiatry and Psychotherapy, Friedrich-Alexander-Universität Erlangen-Nürnberg, University Hospital Erlangen, Erlangen, Germany; ^2^Faculty of Chemistry, Technische Universität Dresden, Dresden, Germany

**Keywords:** amyloid precursor protein, amyloid, Abeta, Alzheimer, cytokine, immune system, secretase, BACE

## Abstract

It has been previously shown that the amyloid precursor protein (APP) support the innate immune defense as an immune receptor. Amyloid β (Aβ) peptides seem to have properties of an antimicrobial peptide and can act as opsonines. In APP-deficient mouse models, a reduced secretion of cytokines has been observed. Still, it is unclear whether this can be attributed to the lack of APP or to the missing secretion of Aβ peptides. We inhibited the secretion of Aβ peptides in primary human monocyte derived macrophages with the γ-secretase inhibitor N-[N-(3,5-Difluorophenacetyl)-L-alanyl]-S-phenylglycine-t-butyl-ester (DAPT) or the β-secretase inhibitor GL-189. Alternatively, we knocked down APP by transfection with siRNA. We measured tumor necrosis factor α (TNFα), interleukin 6 (IL-6) and interleukin (IL-10) by enzyme linked immunosorbent assay (ELISA) and evaluated the phagocytotic activity by flow cytometry. We observed reduced concentrations of TNFα and IL-6 in the media of APP^k/d^ macrophages and after inhibition of the β-, or γ-secretase, especially after additional immunological activation with lipopolysaccharide (LPS). Secretion of IL-10 was increased after pharmacological inhibition of APP processing when the macrophages were not immunologically activated but was decreased during LPS-induced inflammation in APP^k/d^ macrophages. No changes of the phagocytotic activity were observed. We conclude that macrophage APP and Aβ peptides support the initiation of an immune response and are involved in the regulation of TNFα, IL-6, and IL-10 secretion by human monocyte-derived macrophages.

## Background

The amyloid precursor protein (APP) is expressed on nearly every cell type and the amyloid β (Aβ) peptides, which are generated by sequential cleavage of APP by the β- and γ-secretase, are known to aggregate to plaques in the brains of patients with Alzheimer's disease (AD) (1). However, there are individuals with a considerable amount of amyloid plaques who do not show signs of dementia. Furthermore, preventing the agglutination of Aβ peptides in plaques by Aβ-specific antibodies does not stop the progress of dementia (2). Therefore, the causal association of Aβ peptides and Alzheimer's disease may not be as immediate as assumed for the last decades.

Although APP and its cleavage products have been intensely investigated in the context of AD, little is known about their physiological functions and their role within the immune system. Inflammatory processes such as the activation of microglia and peripheral macrophages are increasingly considered in the research of AD pathophysiology (3–5). However, it is still not clear, whether neuroinflammation is the cause or the consequence of AD and whether it is harmful or beneficial (3, 6, 7).

The anti-amyloid antibody Aducanumab was associated with an increased incidence of urinary tract and lung infections in the group with the highest dosage of 10 mg/kg (8). Also, a knock-out of APP or the β-site amyloid cleaving enzyme (BACE-1) in mice was associated with a reduced activity of microglia and a reduced secretion of pro-inflammatory cytokines (9–13). Likewise, reduced concentrations of Aβ peptides in cerebrospinal fluid (CSF) were also found during brain infections (14, 15). One reason for this finding might be that Aβ peptides bind and agglutinate microorganisms and are therefore no longer measurable in the CSF. Astrocytes express higher amounts of the APP processing enzymes BACE-1 and presenilin 1 upon infection with *C. pneumonia*e (16). Therefore, an immunological function of APP and Aβ peptides can be assumed.

Brain microglia and peripheral macrophages both belong to the mononuclear phagocyte system and part of the microglia seems to be recruited from peripheral monocytes transmigrating into the brain (17, 18). Although microglia and peripheral monocyte-derived macrophages differ to some extent, they still share many features (18, 19). As primary human microglia is hard to obtain, monocyte derived macrophages are therefore a frequently used model for certain aspects of microglial biology (19–21). We previously reported that monocytes express APP and that its metabolisation into Aβ peptides depends on their immunological activation (22–24). Phagocytosis of polystyrene particles and *E. coli* was shown to be improved by coating the particles with different Aβ peptide variants (25). Furthermore, an antimicrobial activity of Aβ peptides in cultures of Gram positive and gram negative bacteria as well as *Candida* spp. has been observed (26, 27). Especially the more hydrophobic Aβ peptide variants seem to agglutinate microorganisms and form channels in their cell membranes (27–29). These findings could be confirmed in an *in vivo* model of experimental bacterial meningitis resulting in an improved survival of APP transgenic mice, and a reduced survival of APP^k/o^ mice (30).

The question arises, whether Aβ peptides only support the immune system as opsonin and antimicrobial agent or if they have additional functions as co-stimulatory factors that induce a pro-inflammatory immune response. During inflammation, macrophages secrete a plethora of cytokines (20). Key cytokines indicating a pro-inflammatory reaction are besides others interleukin (IL)-1β, IL-12A, IL-12B, and IL-23, IL-6 and tumor necrosis factor α (TNFα). One of the most important anti-inflammatory cytokines of macrophages is IL-10 (20). We tested, whether the autologous Aβ peptides, secreted by macrophages during inflammatory processes support the immune defense by increasing the secretion of IL-6 and TNFα and by improving the phagocytosis of polystyrene particles.

## Methods

### Preparation and Cultivation of Monocytes

Monocytes were isolated from buffy coats of anonymous healthy erythrocyte donors (Transfusionsmedizin, Suhl, Germany) by density gradient centrifugation and adhesion to polystyrene cell culture dishes in Dulbecco's modified minimal essential medium (DMEM, Pierce biotechnology, Rockford, USA) without serum. As the buffy coats were bought at the blood bank, no ethics approval was necessary. Nine Mio PBMC per well were seeded in a 12-well plate and allowed to adhere for 90 min. Lymphocytes were removed by thorough washing with 4°C Dulbecco's modified phosphate buffered saline (PBS). Cultures only included monocytes of a single donor. All experiments were replicated with the indicated number of donors (biological replicates). Monocytes were then cultivated at 37°C and 5% CO_2_ in Roswell Park Memorial Institute (RPMI) medium (Promocell, Heidelberg, Germany) containing 10% fetal calf serum (FCS, Biochrome, Berlin, Germany) and differentiated into macrophages by adding 40 ng/ml granulocyte-monocyte colony stimulating factor GM-CSF (Immunotools, Friesoythe, Germany). 50% of the medium was exchanged after four days. To avoid interference of endogenous Aβ peptides with those contained in FCS, the medium was changed to serum-free AIM-V medium (Thermo scientific, Dreieich, Germany) at the 7th day *in vitro* (*div*).

An inflammatory reaction was induced either by adding 1 μm polystyrene particles (7/cell) (Polysciences, Hirschberg, Germany) or 10 ng/ml lipopolysaccharide (LPS, Sigma-Aldrich, Munich, Germany) to the cell culture medium at the 9th *div* (secretase inhibitors) or 8th *div* (siRNA transfection), 24 h before measuring cytokine secretion or phagocytotic activity. For a timeline of the experimental procedures see Supplementary Figure 1.

All cell culture experiments were carried out in duplicates and the viability of the cells was assessed with the Cytotox96 non-radioactive assay (Promega, Mannheim, Germany) (Supplementary Figure 2) as well as the (3-(4,5-dimethylthiazol-2-yl)-2,5-diphenyltetrazolium bromide) (MTT)-test according to the manufacturer's instructions (Sigma-Aldrich, Munich, Germany).

### Inhibition of APP Processing

APP processing was pharmacologically inhibited by adding 10 μM of the γ-secretase inhibitor N-[N-(3,5-Difluorophenacetyl)-L-alanyl]-S-phenylglycine-t-butyl-ester (DAPT, Sigma-Aldrich, Munich, Germany) or 500 nM of the tripartite β-secretase inhibitor T_GL−189_ (provided by Prof. Knoelker, Dresden Germany) when exchanging the medium on the 7th *div*. (Supplementary Figure 3) (31, 32). The applied concentration of the secretase inhibitors did not reduce the viability of the cells.

### Transfection of Macrophages

Macrophages were transfected with validated silencer® select siRNA directed toward APP (ID s1500, Thermo Scientific, Dreieich, Germany) using the viromere blue transfection system (Lipocalyx, Halle, Germany) according to the manufacurer's instructions. On the 7th *div*. the medium was exchanged with serum free AIM-V medium. APP siRNA was diluted to 2.8 μM with buffer BLUE. Viromer® BLUE was mixed with buffer BLUE at a ratio of 1:90 and added to the siRNA dilution. After 15 min of incubation, 100 μl of the siRNA mix was added to 1 ml of cell culture medium resulting in a final siRNA concentration of 0.14 μM. Non-silencing silencer® select negative control No. 1 siRNA (Thermo Scientific, Dreieich, Germany) served as control (mock). All experiments were carried out in duplicates.

### Phagocytosis-Assay—Flow Cytometry

To assess the phagocytotic activity of macrophages, fluorescent 1 μm polysterene particles were added in a previously optimized concentration of 20 particles/cell (Supplementary Figure 4). At several timepoints between 10 and 1,200 min, cells were detached with accutase (PAA laboratories, Cölbe, Germany) and the mean fluorescent initensity per macrophage was measured with the CyFlow space flow cytometer (Partec, Goerlitz, Germany) equipped with flow max 2.8 software (Partec, Goerlitz, Germany) and evaluated with the Kaluza 2.0 software (Beckman & Coulter, Krefeld, Germany).

### Enzyme Linked Immunosorbent Assay (ELISA) of TNFα, IL-6, IL-10

The concentrations of TNFα, IL-6, and IL-10 in the conditioned macrophage media were quantified 48 h after transfecting the macrophages with APP siRNA or 72 h after adding the secretase inhibitors by commercially available antibody sets (Catalog numbers: IL-6: 31670069, IL-10: 31670109, TNFα: 31673019; all Immunotools, Friesoythe, Germany). Optimized working concentrations of the respective antibodies were established before the experiments. All measurements were run in duplicates. The samples were diluted to be measured within the detection range of the assays and the coefficient of variation of all measurements was below 20%.

### Immunoprecipitation, Sodium Dodecyl Sulfate Polyacrylamide Gel Electrophoresis (SDS-PAGE), and Immunoblot

The concentrations of APP and Aβ peptides in macrophage cultures were assessed with SDS-PAGE followed by immunoblotting.

For the measurement of APP, cells were lysed with the radioimmunoprecipitation assay (RIPA)-buffer (50 mM 4-(2-hydroxyethyl)-1-piperazineethanesulfonic acid (HEPES), 150 mM NaCl, 1%(v/v) Igepal, 0.5%(w/v) sodiumdeoxycholate, 0.1% SDS and 1 tablet Complete Mini protease inhibitor cocktail (Roche, Germany) per 10 ml. The protein content of cell lysates was assessed with the bicinchoninic acid (BCA)-assay (Pierce Biotechnology, Rockford, USA) and a standardized amount of protein was boiled with sample buffer and loaded on 7,5 % SDS-pages according to Laemmli et al. (33). The consecutive immunoblot on polyvinylidene difluoride (PVDF) membranes was performed according to the method described by Towbin et al. (34). The immunolabeling was carried out with the anti-APP antibody 22C11 (Merck-Millipore, Darmstadt, Germany) followed by incubation with the horseradish peroxidase labeled goat-anti-mouse antibody (Merck-Millipore, Darmstadt, Germany). Membranes were developed with ECL® advance (GE-Healthcare, Freiburg, Germany) and recorded with the Amersham Imager 600 (GE-Healthcare, Freiburg, Germany). A quantification of the blots was performed on the bases of band intensity normalized to the density of the glyceraldehyde 3-phosphate dehydrogenase (GAPDH) band with the quantity one software (Bio-Rad, Munich, Germany).

The concentrations of Aβ peptides in cell culture medium were evaluated according to Wiltfang et al. Aβ peptides were immunoprecipitated with the N-terminal anti-Aβ peptide antibody 1E8 and separated on Tris/Bicine SDS-Pages containing 8 M urea (35). Peptides were transferred to PVDF membranes using a semi-dry westernblot with a discontinuous buffer-system (35). Immunolabeling was performed with the anti-Aβ antibody clone 1E8 and the signal was enhanced by a two-step labeling with a biotinylated goat-anti-mouse antibody and streptavidine conjugated horseradish peroxidase. Finally, membranes were developed with ECL® advance (GE-Healthcare, Freiburg, Germany) and recorded with the Amersham Imager 600 (GE-Healthcare, Freiburg, Germany). Quantification of the blots was carried out with the quantity one software (Bio-Rad, Munich, Germany).

### Statistical Analysis

Statistical analysis was carried out using Prism 6.0 (GraphPad Software Inc., La Jolla, CA, USA). As each experiment was carried out with cells from the same donor, pairwise comparisons were calculated with the ratio paired *t*-test. Results are presented as mean with standard deviations and were considered to be significant at a *p* < 0.05. A *p*-value between 0.05 and 0.1 was referred to as a trend.

## Results

### Reduced Secretion of TNFα and IL-6 After Inhibition of APP Processing

**Figure 1:**
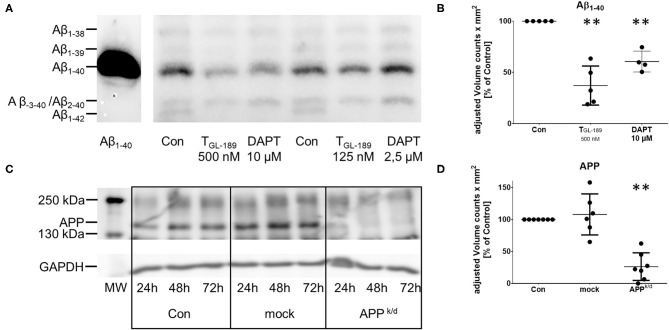
Reduced secretion of Aβ peptides after treatment with T_GL−189_ and DAPT. Reduced expression of APP after knock/down of APP with siRNA. **(A,B)** representative western blot and semiquantitative assessment of the Aβ peptides secreted by primary human macrophages after inhibition of APP processing by the β-secretase inhibitor T_GL−189_ or the γ-secretase inhibitor DAPT. 72 h after the addition of T_GL−189_ or DAPT in the indicated concentrations the media were collected. Cultures treated only with the solvent of the substances (dimethyl sulfoxide, DMSO) served as control (Con). Aβ peptides were analyzed after immunoprecipitation by SDS-Page containing 8 M Urea and subsequent immunoblot. Aβ1-40 was added as a standard. The bands are labeled according to previously published in depth analysis of Aβ peptides secreted by monocytes/macrophages (22–24). According to the results, a concentration of 500 nM for T_GL−189_ and 10 μM for DAPT were chosen for the experiments. **(C,D)** Representative western blot and semiquantitative assessment of mature, fully glycosylated macrophage APP 24, 48, and 72 h after siRNA knock-down of APP. The quantification **(D)** was performed 72 h after the transfection. Transfection of non-coding siRNA served as additional control (mock). The analysis of Aβ and APP was performed at the same time point, when the experiments were carried out. Each point represents the result of a biological replicate. MW, molecular weight marker, **p < 0.01; ***p < 0.001 as compared to control conditions.

Primary human monocyte derived macrophages were cultivated in serum-free media. The secretion of Aβ peptides was inhibited either by addition of the tripartite β-secretase inhibitor T_GL−189_ in a concentration of 500 nM or 10 μM of the γ-secretase inhibitor DAPT. As expected, both treatments reduced the secretion of Aβ_1−40_ and Aβ_1−42_ considerably (Figures 1A,B). The western blot also suggests, that 2.5 μM DAPT does not sufficiently reduce the secretion of Aβ peptides. The amount of Aβ_−3−40_/Aβ_2−40_, which co-migrate in the same lane, remained unchanged as recently described by Oberstein et al. (36). The viability of the cells was not compromised as assessed by measurement of the lactate dehydrogenase (LDH) release into the conditioned media and the reduction of MTT by vital cells.

**Figure 2:**
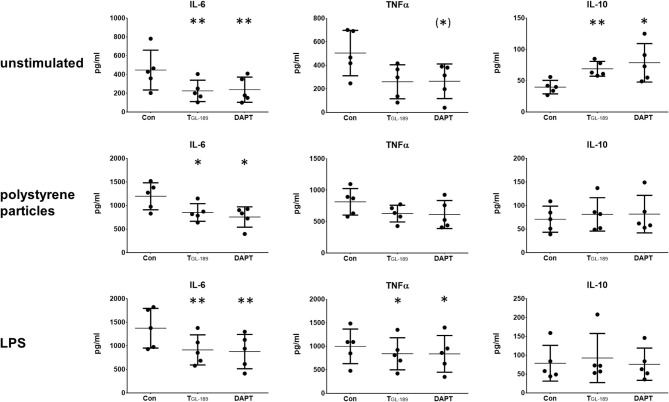
Reduced secretion of IL-6 and TNFα after inhibition of APP processing. IL-6, TNFα, and IL-10 were determined by ELISA in cultures of primary human monocyte derived macrophages (*n* = 5). Cultures were unstimulated (upper row), stimulated with 1 μm polystyrene particles (seven particles/cell) (middle row) or stimulated with 10 ng/ml LPS (bottom row). The secretion of Aβ peptides was inhibited with 500 nM of the β-secretase inhibitor T_GL−189_ or 10 μM of the γ-secretase inhibitor DAPT. Results are presented as mean with standard deviation. ELISA were carried out in duplicates. Each point represents a biological replicate and is the mean value of the duplicates. The significance of the differences was evaluated with the ratio-paired *t*-test between cultures treated with secretase inhibitors and those without. (*) *p* < 0.1 (trend); *p < 0.05; **p < 0.01.

TNFα, IL-6, and IL-10 were determined by ELISA 24 h after the macrophages were immunologically activated either by 10 ng/ml LPS or 1 μm polystyrene particles in a concentration of 7 particles/cell. Reduced concentrations of IL-6 were found 72 h after inhibition of Aβ peptide secretion by T_GL−189_ and DAPT in macrophage cultures without immunological activation as well as in those activated with polystyrene particles or LPS (Figure 2). In cultures activated by LPS T_GL−189_ and DAPT also reduced the concentration of TNFα (Figure 2). Without stimulation and after addition of polystyrene particles, the reduced secretion of TNFα was not statistically significant (*p* = 0.18 and *p* = 0.09, respectively). Interestingly, IL-10 was found elevated after inhibition of APP processing, but only in cultures without an immunological activation (Figure 2). A summary of the results is presented in Table 1.

**Table 1:** Impact of β-/γ-secretase inhibition and APP knockdown on cytokine secretion—summary.

		**IL-6**	**TNFα**	**IL-10**
β-/γ-secretase inhibitor	Con	**↓**	(↓)	**↑**
	polystyrene particles	**↓**	↔	↔
	LPS	**↓**	**↓**	↔
APP^k/d^	Con	↔	↔	↔
	polystyrene particles	↔	↔	↔
	LPS	**↓**	(↓)	**↓**

**Figure 3:**
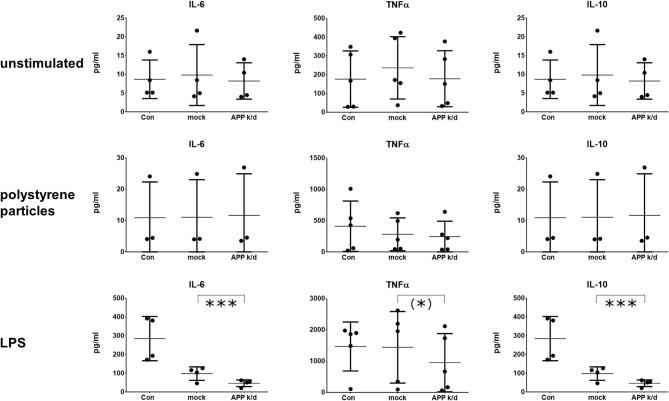
Reduced secretion of IL-6 and IL-10 after knock-down of APP. IL-6 (*n* = 4), TNFα (*n* = 5), and IL-10 (*n* = 4) were determined by ELISA in cultures of primary human monocyte derived macrophages. Cultures were unstimulated (upper row), stimulated with 1 μm polystyrene particles (seven particles/cell) (middle row), or stimulated with 10 ng/ml LPS (bottom row). The expression of APP was inhibited by transfection with siRNA. Cultures transfected with non-coding siRNA served as control (mock). Results are presented as mean with standard deviation. ELISA were carried out in duplicates. Each point represents a biological replicate and is the mean value of the duplicates. Significance of the differences was evaluated with the ratio-paired *t*-test between mock-transfected and APP-transfected macrophages. (*) *p* < 0.1 (trend); ***p < 0.001.

*The table summarizes the data presented in Figures 2, 3. **↓** significant reduction of cytokine secretion; **↑** significant increase of cytokine secretion; (↓) trend for a reduced secretion of cytokine; ↔ no change of cytokine secretion*.

### Reduced Secretion of IL-6 and IL-10 After Inhibition of APP Expression

To discriminate the impact of the APP from Aβ peptides, the expression of APP was inhibited by a siRNA knock-down of APP in the same macrophage cultures. Transfection with a non-binding siRNA (mock) served as control and viability was tested as indicated above (Supplementary Figure 2). The reduced concentration of APP in cell lysates 72 h after the transfection is shown in Figures 1C,D. The medium remained on the cells for 24 h, 48 h after the transfection giving 72 h of incubation with siRNA. The knock-down of APP reduced the concentration of IL-6 and TNFα (trend) in the media of LPS activated macrophages (Figure 3). Unexpectedly, the secretion of IL-6 and IL-10 was also reduced after transfection with non-binding siRNA. However, the effect of the transfection with siRNA directed toward APP was significantly stronger than that of the transfection with non-binding siRNA. The transfection with APP siRNA did not change the cytokine secretion in cells that were unchallenged or activated by phagocytosis of polystyrene particles (Figure 3). While the pharmacological inhibition of APP processing resulted in increased concentrations of IL-10 in LPS activated cultures, the knock down of APP reduced the concentration of IL-10 in the medium (Figure 3). Again, no change of IL-10 was found in unchallenged or particle-challenged cultures of APP^k/d^ macrophages (Figure 3). A summary of the results can be found in Table 1.

### No Change in the Phagocytic Activity of Monocytes After Inhibition of APP Processing or Knock-Down of APP

**Figure 4:**
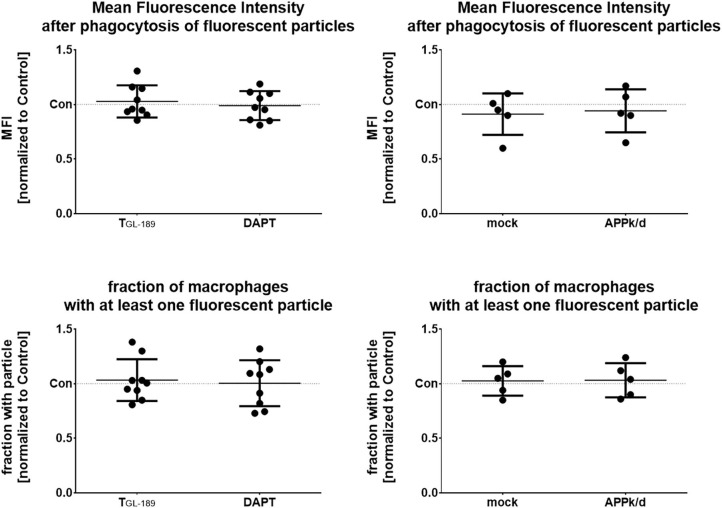
No change of the phagocytotic activity after inhibition of APP processing or knock-down of APP. Macrophages were treated with 500 nM of the β-secretase inhibitor T_GL−189_ or 10 μM of the γ-secretase inhibitor, DAPT (*n* = 9). Alternatively, APP was knocked-down by siRNA (*n* = 5). Phagocytotic activity of the macrophages was determined by flow cytometry 240 min after addition of fluorescent 1 μm polystyrene particles. Results are presented as mean with standard deviation of the measured mean fluorescence intensity (MFI) of the macrophages after phagocytosis. Measurements were carried out in duplicates. Each point represents a biological replicate and is the mean value of the duplicates. Phagocytosis was also evaluated by comparing the fraction of macrophages that contained at least one fluorescent particle normalized in the same way.

To evaluate the impact of APP expression and Aβ peptide secretion on phagocytosis, APP processing was either pharmacologically inhibited or APP was knocked down by siRNA as detailed above. After establishing the optimal concentration of fluorescent particles and time of measurement, phagocytosis was determined by flow cytometry 240 min after adding fluorescent 1 μm microparticles (20 particles/cell) to the cultures (Supplementary Figure 4). However, neither the inhibition of the β- or γ-secretase nor the APP knock-down affected the amount of intracellular particles as indicated by the mean fluorescent intensity (MFI) or the fraction of macrophages that is associated with at least one fluorescent particle (Figure 4).

## Discussion

We showed that the pharmacological inhibition of APP processing by the tripartite β-secretase inhibitor T_GL−189_ and an established γ-secretase inhibitor (DAPT) reduced the secretion of IL-6 and increased the anti-inflammatory IL-10 in primary human monocyte-derived macrophages of healthy donors. During LPS induced inflammation, reduced concentrations of IL-6 and TNFα were observed. After an APP knock/down, IL-6 and IL-10 were reduced in macrophages which were activated by LPS.

Interestingly, the changes of cytokine expression induced by APP knockdown and Inhibition of APP processing differ from each other. APP knockdown lead to reduced cytokine secretion only after stimulation with LPS. After pharmacological inhibition of the generation of amyloidogenic Aβ peptides, the secretion of IL-6 and TNFα was reduced under all conditions, however, for TNFα the effect was only significant after stimulation with LPS. Therefore, it seems that IL-6 is stronger affected by alterations in the APP metabolism than TNFα and IL-10. Additionally, the effects produced by β-/γ-secretase inhibition and APP knockdown seem to be increased under inflammatory conditions induced by LPS. This could explain why we do only see non-significant reductions of TNFα after β-/γ-secretase inhibition under control conditions and stimulation with polystyrene particles. And it could also explain, why we see changes of cytokine secretion after APP knockdown only after stimulation with LPS.

A very interesting finding is the increased secretion of IL-10 in unstimulated cultures after the inhibition of APP processing. This increase is not visible after stimulation with polystyrene particles and LPS, probably, because the anti-inflammatory effect by lowering the Aβ peptide production is superimposed by the phagocytic and inflammatory challenge. In contrast, IL-10 is reduced after APP knockdown and stimulation with LPS. This indicates that the reduction of Aβ peptides has an anti-inflammatory effect whereas the reduction of APP expression reduces the secretion of pro- and anti-inflammatory cytokines. An explanation for this difference could be that APP has functions as an immune receptor (11). Therefore, the knockdown of APP does inhibit the generation of Aβ peptides but also reduces the expression of a cellular receptor for immunological signals. The consequence is that the macrophage cannot adequately react to the pro-inflammatory activation with LPS, resulting in an impaired secretion of all cytokines including IL-10.

A major limitation of this work is the incomplete inhibition of APP expression and processing. Neither the pharmacological inhibition of APP cleavage nor the knockdown of APP resulted in a complete absence of Aβ peptides or APP, respectively. This probably leads to a considerable underestimation of the effects. The reasons for this are a limitation of inhibitor concentrations by toxicity and unwanted side-effects as well as the existence of additional β- and γ-secretases not inhibited by the applied substances such as meprin-β or cathepsin B (36–38). Primary human macrophages are, besides neurons, probably the most difficult cells for transfection experiments. Therefore, several different techniques including lipofection and electroporation have been tested and rejected, before the transfection with viromers lead to acceptable results. A rate of transfected cells of about 80% was measured by transfection with fluorescent siRNA and stealth siRNA. The transfection with siRNA directed at APP reduced the expression of APP to ~25% in our experiments. Interestingly, the transfection with non-binding siRNA, meant as a control, did reduce the secretion of IL-6 and IL-10 in macrophage cultures activated with LPS. This effect was reproducible with a second non-binding siRNA and was not caused by reduced viability of the cells. However, we are currently not able to explain this finding.

To increase the probability of our reported findings not being due to pharmacological side effects, we used two different substances (T_GL−189_ and DAPT) with two different mechanisms (inhibition of β- and γ-secretase). It was described previously that the application of GL-189 as a tripartite substance (T_GL−189_) reduces unspecific side effects by directing the pharmacophore to the catalytic center of the β-secretase (31, 32, 36). The reported reduction of IL-6 and TNFα as well as the increased secretion of IL-10 are therefore very probable induced by the reduced production of Aβ peptides.

Blockage of the β-secretase pathway normally increases processing via the α-secretase pathway, resulting in increased concentrations of sAPPα (31). While we have not measured sAPPα, our results still suggest that macrophage sAPPα is not able to replace the missing Aβ peptides. This is opposing earlier publications, which found that sAPPα activates microglia (39–41). However, this discrepancy might be an issue of concentration and the impact of amyloid peptides was not assessed in former experiments.

It is long known that Aβ fibrils and oligomers activate macrophages and microglia (41, 42). However, our data suggests that not only external Aβ but also the Aβ peptides produced by macrophages themselves have an activating effect on the secretion of pro-inflammatory cytokines. As a consequence, the missing ability to produce Aβ peptides impaired the pro-inflammatory reaction induced by LPS. We and others previously reported that the expression of APP and the secretion of Aβ peptides by monocytes/macrophages depends on their immunological activation (22, 24, 43). Expression of APP and secretion of Aβ peptides was increased during phagocytosis and LPS-induced inflammation. In this context it seems possible that the Aβ peptides are part of a self-energizing circuit initiating an immune response.

Further functions of Aβ peptides within the immune defense as antimicrobial agent and opsonine have been shown (26, 27, 30). The reason, why an inhibition of Aβ peptide generation had no impact on phagocytosis although it changed the concentrations of IL-6, TNFα, and IL-10 in this study might be that phagocytosis is strongly affected by opsonines and the expression of receptors involved in phagocytosis but poorly by the investigated cytokines (44). Furthermore, the observed changes in cytokine levels after stimulation with polystyrene particles are in a 10–20% range. Probably the error of measurement in the phagocytosis assay is too high to detect such subtle changes in macrophage activation. Effects caused by Aβ peptides as an opsonine could probably not be seen in this study because the changes in Aβ peptide concentrations were too small to induce a measurable effect. When describing an opsonizing activity of Aβ peptides, Condic et al. used Aβ peptide concentrations of 1 mg/ml for the opsonization (25). The change in Aβ peptide concentration in our experiments was below 1 μg/ml.

Kumar and his colleagues demonstrated that APP knockout mice had a reduced survival, while mice transgenic for APP had an improved survival in a model of infectious meningitis (30). Fitting into this hypothesis, an increased expression of APP, an accumulation of Aβ peptides in the brain and reduced concentrations of Aβ peptides in the CSF were not only observed in patients with Alzheimer's disease but also with meningitis and other inflammatory diseases (14, 15, 45–47).

Regarding AD this would indicate, that the Aβ peptide deposition could be the consequence and not the cause of neuroinflammation. This idea is supported by epidemiological data showing a reduced risk of AD in patients using non-steroidal anti-inflammatory drugs (48). TNFα antagonists also seem to improve cognitive performance in AD patients (49, 50). Some even hypothesize an infectious agent as the cause of AD (51–55).

Pharmacological inhibition of Aβ peptide generation reduced Aβ_1−x_ but not N-terminal modified Aβ peptides. This indicates, that the Aβ_1−x_ species are responsible for the observed differences. As we did not analyze the aggregation state of the Aβ peptides in our cultures we are not able to differentiate whether Aβ monomers, oligomers or fibrils are responsible for the observed effects. However, Aβ aggregation takes place within few hours and aggregation of Aβ peptides in cultures of macrophages has been shown (56). Therefore, it seems very likely, that at least part of the secreted Aβ peptides aggregate to oligomers and fibrils. Several receptors expressed by macrophages have been shown to bind Aβ peptide fibrils or oligomeres, [e.g., CD14, CD36, macrophage scavenger receptor 1, N-formyl-peptide receptor like-1 and APP (11, 57)]. Binding of these receptors triggers downstream thyrosin kinases, release of Ca^++^ and ultimately activation of NFkB and CREB (41, 57–64).

In microglial cultures of APP knock-out mice as well as in brains and intestines of these mice a reduced motility of macrophages as well as reduced concentrations of several cytokines, including IL-6, TNFα, and IL-10 were observed which is in accordance to our findings (9–11). However, due to their methodology, the authors could not discriminate between the effects caused by APP and those caused by Aβ peptides. Consequently, they discuss the role of APP as a receptor for LPS or a transcription factor. The different effects of the APP knock-down and pharmacological inhibition of APP processing concerning the IL-10 concentrations after stimulation with LPS support this assumption. Pro- and anti-inflammatory activities are reduced in APP^k/o^/APP^k/d^ macrophages. When APP as a cell bound protein remains intact, the pharmacological inhibition of Aβ peptide generation removes a pro-inflammatory peptide and might result in a more anti-inflammatory state of the macrophages with reduced secretion of IL-6 and increased secretion of IL-10.

## Conclusion

Taken together, the presented data supports the hypothesis that APP and Aβ peptides expressed and secreted by macrophages are involved in initiating and regulating immune responses in healthy donors. Further studies are necessary to see if this is also the case for individuals suffering from Alzheimer's disease. In clinical trials testing Aβ lowering therapies, dysfunctions of the immune system should be closely monitored.

## Data Availability Statement

The datasets generated for this study are available on request to the corresponding author.

## Author Contributions

PS, MW, CG, TO, JK, and JM designed the study. PS, MW, and CG carried out the experiments and statistics. PL and H-JK developed and provided the tripartite β-secretase inhibitor. Data was analyzed and evaluated by PS, MW, CG, TO, JK, and JM. PS, MW, and JM drafted the manuscript. All authors critically reviewed the manuscript, provided constructive comments to improve the quality of the manuscript, read, and approved the final manuscript. All authors contributed to the article and approved the submitted version.

## Conflict of Interest

The authors declare that the research was conducted in the absence of any commercial or financial relationships that could be construed as a potential conflict of interest.

## Supplementary Material

Supplementary Figure 1 Timeline of experimental procedures.

Supplementary Figure 2 No reduction of macrophage viability by the inhibitors of APP metabolism and transfection.

Supplementary Figure 3 Structure of the tripartite ?-secretase inhibitor TGL-189.

Supplementary Figure 4 Time- and dose-dependency of phagocytosis.

## Abbreviations

APP: Amyloid precursor protein
Aβ: Amyloid-β
AD: Alzheimer's disease
BACE: Beta site amyloid cleaving enzyme
BCA: Bicinchoninic acid
CSF: Cerebrospinal fluid
DAPT: N-[N-(3,5-Difluorophenacetyl)-L-alanyl]-S-phenylglycine-t-butyl-ester
*div*: Days *in vitro*
DMEM: Dulbecco's modified minimal essential medium
DMSO: Dimethyl sulfoxide
ELISA: Enzyme linked immunosorbent assay
GAPDH: Glyceraldehyde 3-phosphate dehydrogenase
GM-CSF: Granulocyte-monocyte colony stimulating factor
HEPES: 4-(2-hydroxyethyl)-1-piperazineethanesulfonic acid
IL-6: Interleukin 6
IL-10: Interleukin 10
LDH: Lactate dehydrogenase
LPS: Lipopolysaccharide
MFI: mean fluorescent intensity
MTT: (3-(4,5-dimethylthiazol-2-yl)-2,5-diphenyltetrazolium bromide)
RIPA: Radioimmunoprecipitation assay
RPMI: Roswell Park Memorial Institute
PBS: Phosphate buffered saline
PVDF: Polyvinylidene difluoride
SDS-PAGE: Sodium dodecyl sulfate polyacrylamide gel electrophoresis
TNFα: Tumor necrosis factor α.
