# Supplementary figures and images for: Pharmacological Inhibition of Amyloidogenic APP Processing and Knock-Down of APP in Primary Human Macrophages Impairs the Secretion of Cytokines
Source: Front Immunol. 2020 Sep 3;11:1967. doi: 10.3389/fimmu.2020.01967 (PMC7494750; doi:10.3389/fimmu.2020.01967)

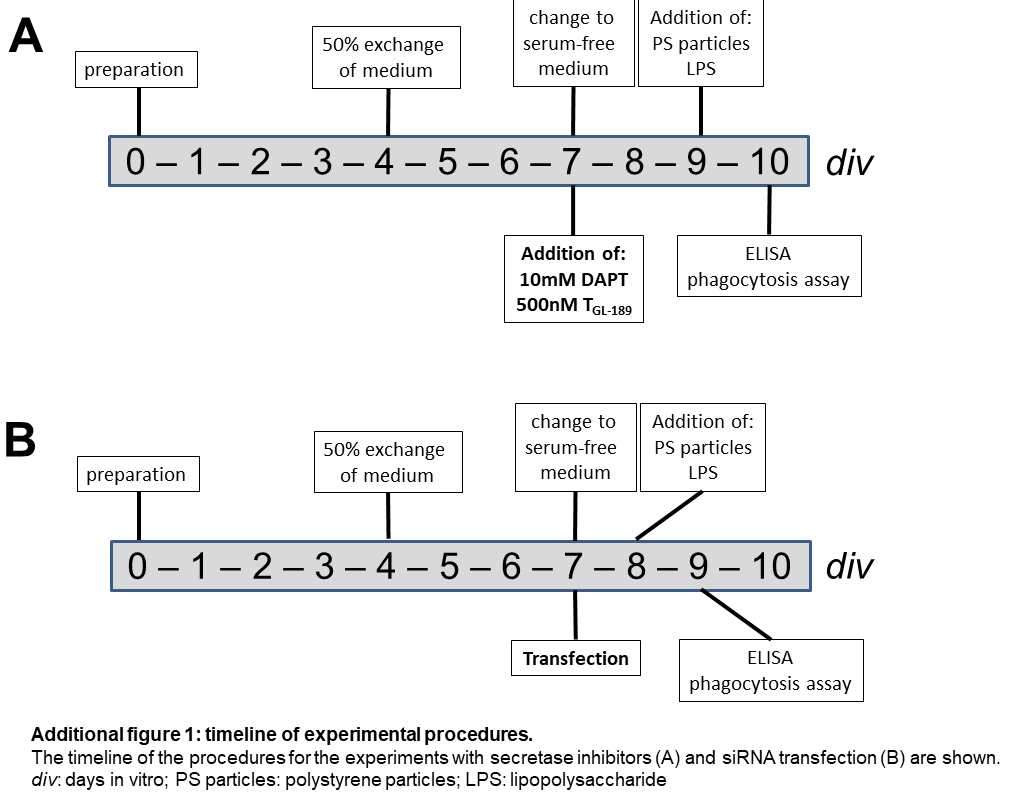

Supplement: Supplementary Figure 1 — Timeline of experimental procedures. [file Image_1.tif]

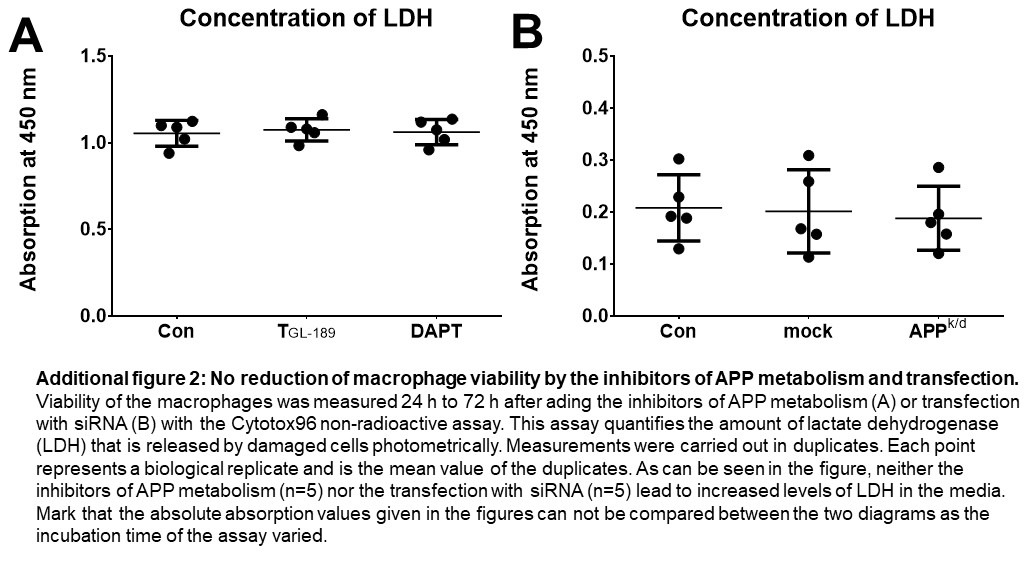

Supplement: Supplementary Figure 2 — No reduction of macrophage viability by the inhibitors of APP metabolism and transfection. [file Image_2.TIF]

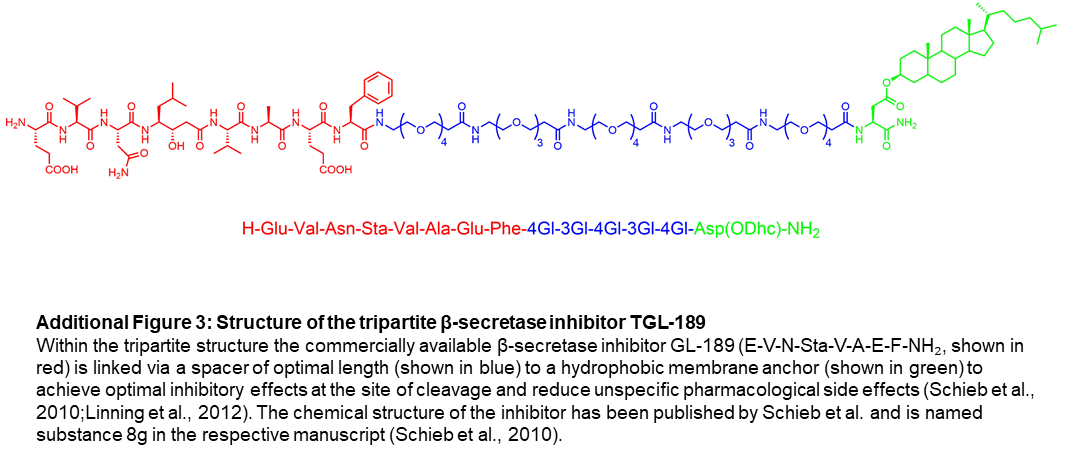

Supplement: Supplementary Figure 3 — Structure of the tripartite ?-secretase inhibitor TGL-189. [file Image_3.TIF]

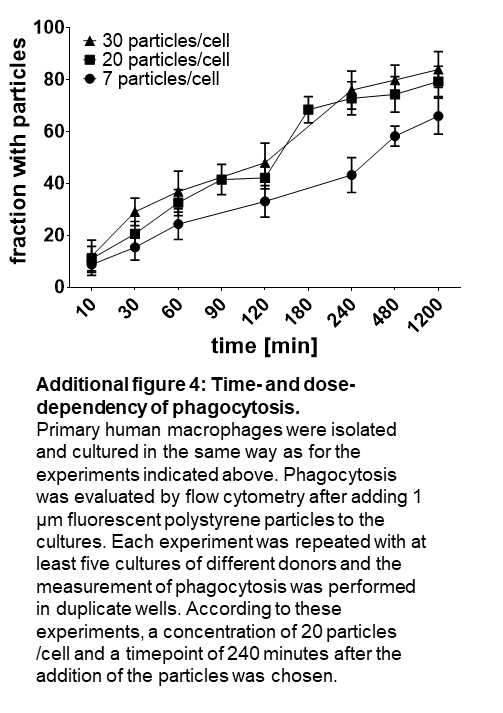

Supplement: Supplementary Figure 4 — Time- and dose-dependency of phagocytosis. [file Image_4.TIF]
